# Atretic Double Aortic Arch: Imaging Appearance of a Rare Anomaly and Differentiation From Its Mimics

**DOI:** 10.7759/cureus.9478

**Published:** 2020-07-30

**Authors:** Sarv Priya, Prashant Nagpal

**Affiliations:** 1 Radiology, University of Iowa Hospitals and Clinics, Iowa City, USA; 2 Cardiothoracic Radiology, University of Iowa Hospitals and Clinics, Iowa City, USA

**Keywords:** atretic double aortic arch, double aortic arch mimics, vascular ring, right aortic arch, cta double aortic arch, virtual reality, four vessel sign, congenital aortic arch anomaly

## Abstract

A double aortic arch (DAA) with atresia is an uncommon cause of a symptomatic vascular ring resulting in trachea-esophageal compression. An atretic double aortic arch can resemble the right aortic arch with a mirror image branching pattern or the right arch with an aberrant left subclavian artery depending upon the level of atresia. The double aortic arch with atresia is difficult to detect on pre-surgical computed tomography angiography or magnetic resonance angiography due to a lack of contrast in the obliterated arch segment. Differentiation of a double arch with atresia from the right aortic arch is vital as an atretic double arch is a form of the complete vascular ring while the right aortic arch may or may not be symptomatic. Knowledge of some key imaging features can help distinguish these entities. In this case report, we discuss an uncommon case of a double aortic arch with atresia between the left common carotid and left subclavian artery. We also describe its close mimics, their embryological basis, and ways to differentiate it from the right aortic arch.

## Introduction

Vascular rings are a rare form of aortic arch anomaly resulting in trachea-esophageal compression and represent 1%-3% of all congenital heart diseases [1]. Double aortic arch (DAA) constitutes about 46%-76% of all symptomatic congenital vascular rings [2]. DAA with atresia is an even rarer cause of a complete vascular ring and is difficult to detect on pre-surgical computed tomography angiography (CTA) or magnetic resonance angiography (MRA) imaging due to lack of contrast in the obliterated atretic segment [3]. An atretic double arch can mimic the right-sided aortic arch with mirror image branching or right arch with aberrant left subclavian artery depending upon the level of atresia. In this work, we highlight a rare case of DAA with atresia and discuss approaches to accurately characterize the atretic double aortic arch and describe diagnostic clues to differentiate it from mimics of the right aortic arch.

## Case presentation

**Figure 1:**
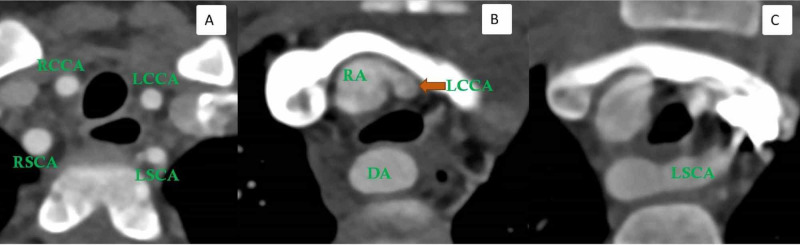
Double aortic arch with atresia between the left common carotid and the left subclavian artery in a two-year-old male child Axial CTA image (A) shows four aortic branches in a relatively symmetrical presentation seen just above the aortic arch (four-vessel sign). Axial CTA image (B) shows the posterior course and tenting of the left common carotid artery (arrow). Axial CTA image (C) shows the left subclavian artery coursing behind the esophagus and coursing from right to left. RSCA: Right subclavian artery; LSCA: Left subclavian artery; RCCA: Right common carotid artery; LCCA: Left common carotid artery; DA: Descending aorta; RA: Right arch; CTA: Computed tomography angiography; MRA: Magnetic resonance angiography

A two-year-old male child with a history of noisy breathing presented with a concern of murmur heard on clinical examination. A grade I/II murmur was heard on the left sternal border. Transthoracic echocardiogram was limited due to a lack of patient cooperation with incomplete visualization of the aortic arch but showed mild flow turbulence in the aortic arch and dampened flow in the descending thoracic aorta. Pulsatile flow was seen to the left of the left pulmonary artery that could not be fully evaluated on echocardiogram. The child underwent computed tomography angiography (CTA) of the chest for further evaluation. Non-gated CTA of the chest (DLP 43 mGy*cm) was performed in high-pitch (FLASH) mode on the Siemens dual-source scanner (Siemens Drive, Erlangen, Germany). CT showed a relatively symmetrical appearance of both common carotids and subclavian arteries (four-vessel sign) just above the level of the aortic arch (Figure 1A). There was evidence of a posterior course with mild tethering of the left common carotid artery (LCCA) at its origin (first branch of the aortic arch) (Figure 1B). The left subclavian artery (LSCA) was seen coursing behind the esophagus (Figure 1C) with the right-sided descending aorta (Video 1).

**Video 1:** Double aortic arch with atresia between the left common carotid and the left subclavian artery in a two-year-old male child Axial cine image of a patient with a double aortic arch and atresia between the left common carotid and left subclavian arteries shows a four-vessel sign and the posterior course of the left common carotid artery

**Figure 2:**
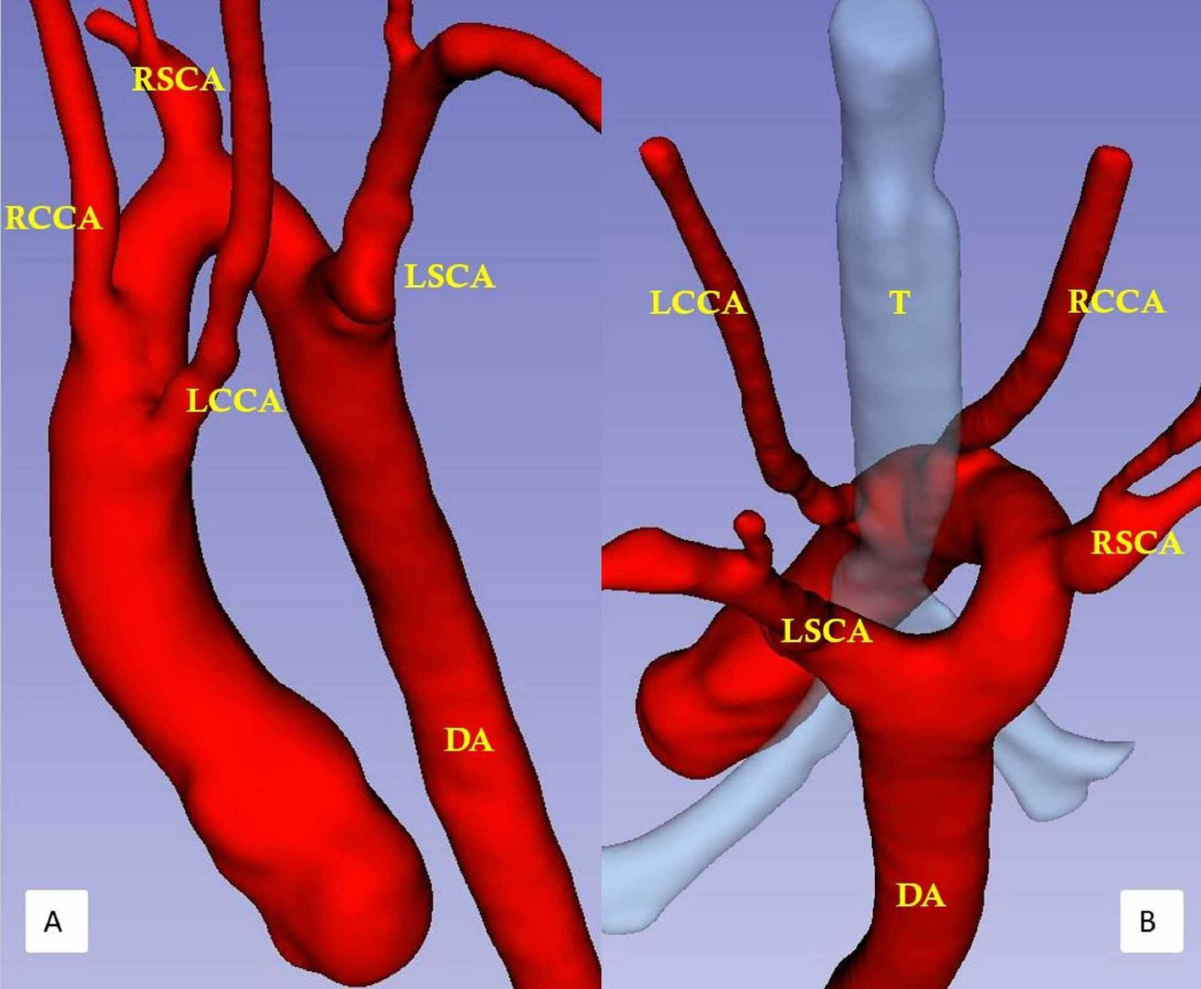
Virtual reality model of a patient with a double arch and atresia between the left common carotid artery and the left subclavian artery Virtual reality model in lateral (A) and posterior (B) views shows the left CCA as the first branch, followed by the right CCA, right SCA, with left SCA as the last branch seen in the retrotracheal course. RSCA: Right subclavian artery; LSCA: Left subclavian artery; RCCA: Right common carotid artery; LCCA: Left common carotid artery; DA: Descending aorta; T: Trachea

The reconstructed three-dimensional model (Interactive Model 1) showed the absence of the aortic segment between the left CCA and left SCA and helped in a clear understanding of the anatomy and for pre-surgical planning (Figures 2A-2B).

**Video 2:** Atretic double aortic arch Double aortic arch with atresia between the left common carotid and the left subclavian artery in a two-year-old male child

The presence of symmetric four-vessel aortic arch branches, the posterior course of the first aortic arch branch (LCCA), and diverticular outpouching from the descending thoracic aorta helped suggest that imaging was highly suggestive of DAA with atresia between the LCCA and LSCA. The vascular ring was completed by the dominant aortic arch on the right, ascending aorta anteriorly, retroesophageal aortic segment posteriorly, and an atretic segment of the left aortic arch on the left side. Due to the concern of complete vascular ring and symptoms, the child underwent surgical repair via left thoracotomy, and an obliterative segment was found between LCCA and LSCA, confirming the atresia as seen on CTA chest. The child was successfully treated by the division of the atretic left-sided aortic arch.

## Discussion

**Figure 3:**
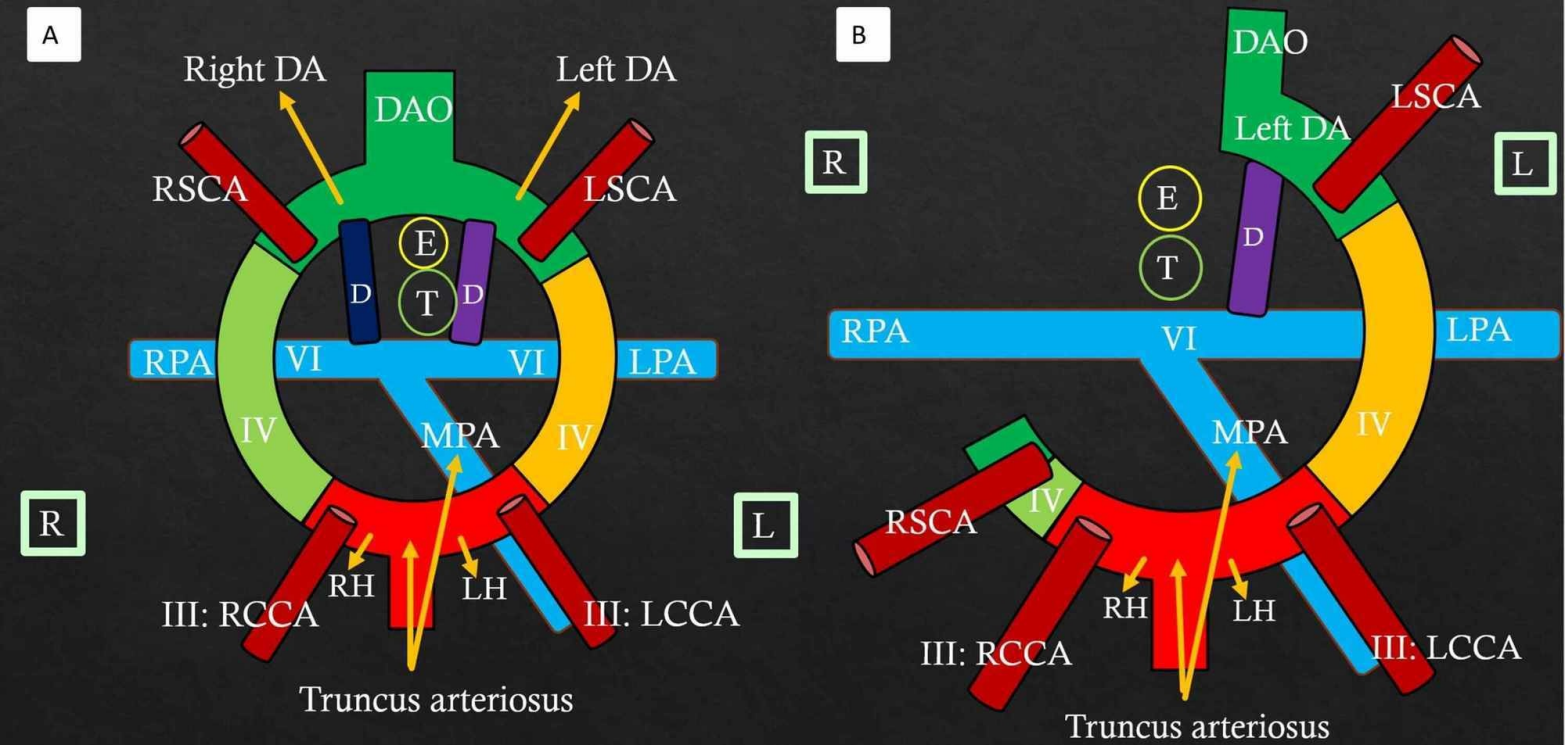
Edwards' hypothetical double aortic arch model and normal left aortic arch formation Color diagram (A) shows the components of a double aortic arch. III, IV, and VI refer to the third, fourth, and sixth branchial arches on both sides. RH: Right horn of aortic sac; LH: Left horn of aortic sac; RSCA: Right subclavian artery; LSCA: Left subclavian artery; MPA: Main pulmonary artery; RPA: Right pulmonary artery; LPA: Left pulmonary artery; RCCA: Right common carotid artery; LCCA: Left common carotid artery; Right DA: Right dorsal aorta; Left DA: Left dorsal aorta; DAO: Unpaired fused dorsal aorta; D: Ductus; T: Trachea; E: Esophagus. R and L in square boxes mention right and left orientation Color diagram (B) shows the formation of a normal left aortic arch. Truncus arteriosus gives rise to the ascending aorta and main pulmonary artery. A small segment of the right fourth arch (light green) remains patent and will later form part of the right subclavian artery. The left fourth bronchial arch (yellow) forms the normal left aortic arch. There is a dissolution of the right sixth arch (ductus) and right dorsal aorta distal to the right subclavian artery. The left descending aorta and fused unpaired dorsal aorta (dark green) persist. The third arch forms the common carotid and proximal internal carotid arteries. The left subclavian artery is formed from the left seventh intersegmental artery. The right and left pulmonary arteries arise from the ventral portion of the sixth aortic arch. The left sixth arch forms the left ductus (purple). RH: Right horn of aortic sac; LH: Left horn of aortic sac; RSCA: Right subclavian artery; LSCA: Left subclavian artery; MPA: Main pulmonary artery; RPA: Right pulmonary artery; LPA: Left pulmonary artery; RCCA: Right common carotid artery; LCCA: Left common carotid artery; Left DA: Left dorsal aorta; DAO: Unpaired fused dorsal aorta; D: Ductus; T: Trachea; E: Esophagus. R and L in square boxes mention right and left orientation

The embryological basis of normal aortic arch formation is critical to understand the complex arch anomalies. Edwards’ hypothetical DAA model helps better understand the aortic arch malformations (Figures 3A-3B) and has been previously described in detail [4].

DAA is formed when there is the persistence of the bilateral fourth branchial arches, one on each side of the trachea and the esophagus. Each aortic arch gives rise to the respective right and left common carotid and subclavian arteries. Both arches join to form a single descending aorta, which is commonly located to the left of the spine. Less often, the descending aorta is seen on the right of the spine and rarely is midline in location. The ductus, if present, is left-sided but rarely can be right-sided or bilateral [5]. Clinically, patients present early with stridor, coughing, wheezing, or feeding difficulties if both arches are patent [6]. In patients with loose vascular ring or atresia of one arch, the symptoms can be delayed, or sometimes patients can be asymptomatic. In general, DAA is not associated with other congenital cardiovascular diseases [7]. However, rarely, there may be associated cardiac malformations like tetralogy of Fallot, truncus arteriosus, or transposition of great arteries [8].

Imaging

Echocardiography is often the initial investigation performed for the evaluation of suspected vascular ring. However, it is operator dependent and is often limited if the child is small and uncooperative [6]. Catheter angiography is invasive and is also limited due to only being two-dimensional. Cross-sectional imaging modalities, including computed tomography angiography (CTA) or magnetic resonance angiography (MRA), are excellent for the evaluation of patients with a suspected double arch [4,9]. Both CTA and MRA have their advantages and disadvantages, which have been discussed extensively in the literature [4,10]. High-pitch, non-gated CTA chest can be performed for the evaluation of the vascular ring and provides high-resolution images without the need of electrocardiogram-gating and hence minimum radiation dose [11]. Three-dimensional reconstruction provides a detailed assessment of the aortic arch and its branches and relationship with surrounding structures. Virtual reality modeling is a relatively new technique and the models can be created from thin-slice CT data, allowing excellent depth perception with an improved understanding of the arch spatial relationship with adjacent structures in patients with complex anatomy [12-14]. Besides, CTA allows the simultaneous evaluation of airways for the degree of narrowing and planning an airway intervention.

Double aortic arch without or with atresia

In the majority of patients, both aortic arches are patent, and CTA with three-dimensional reconstruction can easily visualize a double aortic arch if both arches are patent. In general, the right aortic arch is dominant and is cephalic relative to the left arch. However, both arches may be equal in caliber, and less commonly, the left arch may be dominant [15].

Rarely, there may be atresia of a segment of one aortic arch, which most commonly involves the left side [16]. The atretic segment is replaced by a fibrous cord. Accurate diagnosis of a double aortic arch with an atretic segment is critical for timely diagnosis and management, as undiagnosed and unrepaired DAA may result in respiratory compromise later. CT or MRA visualization of an atretic segment is almost impossible due to the absence of contrast in the obliterated fibrotic lumen. Thus, looking for secondary diagnostic clues are essential to characterize the DAA with suspected atresia.

**Figure 4:**
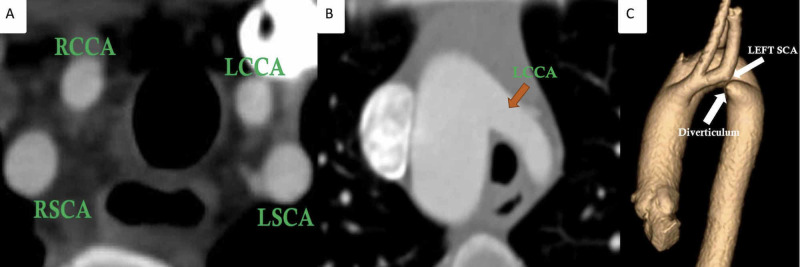
Typical imaging clues of the atretic double aortic arch Axial image (A) showing the symmetrical distribution of common carotid and subclavian arteries (four-vessel sign) seen just above the aortic arch in a patient with a double arch with atresia. Evidence of the posterior course of a patent initial segment of the atretic left arch (B) as shown by the posterior course of the left common carotid artery (LCCA, arrow in B) suggests a double aortic arch with atresia. Evidence of an anteriorly directed diverticulum arising from the descending aorta as seen in the shown volume-rendered image (C) with posterior kinking of the left subclavian artery suggests a connection between a patent segment of the atretic left arch and descending aorta. RSCA: Right subclavian artery; LSCA: Left subclavian artery; RCCA: Right common carotid artery; LCCA: Left common carotid artery

On axial CTA images, the presence of four aortic arch branches (right common carotid, right subclavian, left common carotid, and left subclavian artery) in a symmetric trapezoidal fashion seen just above the level of the aortic arch is a highly sensitive sign of DAA (Figure 4A) [17].

Secondly, the first aortic branch, as seen on axial images, is more posterior in its initial course in patients with atretic DAA (Figure 4B). Tethering or posterior tenting of the patent segment of the left arch with diverticular outpouching from the descending aorta (Figure 4C, Video 2) is another important marker of an atretic segment between an incomplete left arch and the descending aorta [18].

**Video 3:** Volume rendering in a patient with double arch and atresia distal to the left subclavian artery Volume rendered image in a patient with a double arch and atresia distal to the left subclavian artery shows posterior kinking of the left subclavian artery. Also seen is diverticular outpouching from descending aorta directed anteriorly towards the left subclavian artery suggesting evidence of a connection between them.

**Figure 5:**
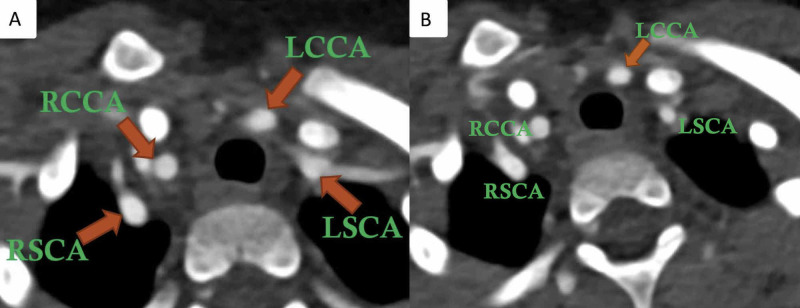
Right aortic arch branching pattern Axial CTA (A) image in a patient with a right aortic arch and mirror-image branching shows a non-symmetrical arrangement of aortic branches. Axial CTA image in the same patient as (A) shows the anterior course of the left common carotid artery. RSCA: Right subclavian artery; LSCA: Left subclavian artery; RCCA: Right common carotid artery; LCCA: Left common carotid artery

In contrast, in the right aortic arch, no symmetric pattern of arch branches is seen (Figure 5A) with the anterior course of the first branch of the aortic arch (Figure 5B) [19].

Mimics of the atretic double aortic arch

DAA With Atresia Distal to the Left Subclavian Artery

**Figure 6:**
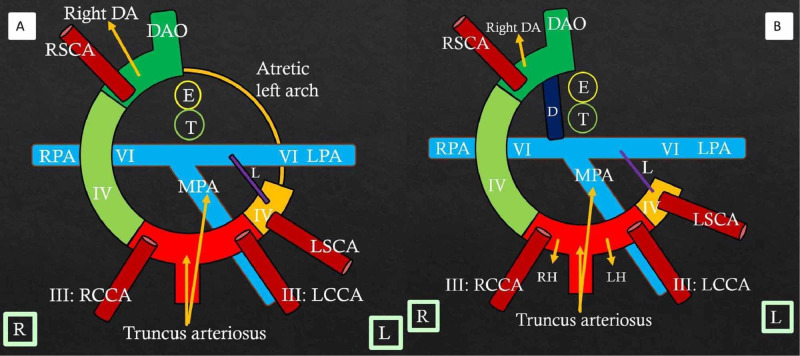
Double aortic arch with atresia distal to the left subclavian artery: Mimics the right aortic arch with Type 1 mirror image branching Color diagram (A) shows atresia of the left fourth aortic arch (yellow) distal to the left ligament arteriosum and the left subclavian artery, with the ligament connecting anteriorly to the left arch. Here, the ductus is directed anteriorly. This appearance resembles the right aortic arch with Type 1 mirror image branching (B) due to non-visualization of the atretic arch segment on CTA. Color diagram (B) shows the right arch with Type 1 mirror-image branching in which there is the dissolution of the left fourth arch and left dorsal aorta with persistence of the right ductus. A small segment of the left fourth arch persists that forms part of the left subclavian artery. Ductus (L) is directed anteriorly with no vascular ring formation. RH: Right horn of aortic sac; LH: Left horn of aortic sac; RSCA: Right subclavian artery; LSCA: Left subclavian artery; MPA: Main pulmonary artery; RPA: Right pulmonary artery; LPA: Left pulmonary artery; RCCA: Right common carotid artery; LCCA: Left common carotid artery; Right DA: Right dorsal aorta; Left DA: Left dorsal aorta; DAO: Unpaired fused dorsal aorta; D: Ductus; T: Trachea; E: Esophagus; L: Ligamentum arteriosum. R and L in square boxes mention right and left orientation

In these patients, the atretic DAA (Figure 6A, Video 3) mimics the right aortic arch with a Type 1 mirror image branching pattern (Figure 6B, video 4) [4].

**Video 4:** Double arch with atresia distal to the left subclavian artery Axial cine image in a patient with a double aortic arch and atresia distal to the left subclavian artery shows a relatively symmetric four vessel sign and posterior course of the patent segment of the atretic left arch.

**Video 5:** Type 1 right aortic arch - mirror image branching Axial cine image in a patient with a right aortic arch and type 1 mirror image branching shows asymmetric arch branching pattern unlike the four-vessel symmetry of the atretic double arch. Also seen is the anterior course of the left brachiocephalic artery.

DAA With Atresia Between the Left Common Carotid and Left Subclavian Arteries

**Figure 7:**
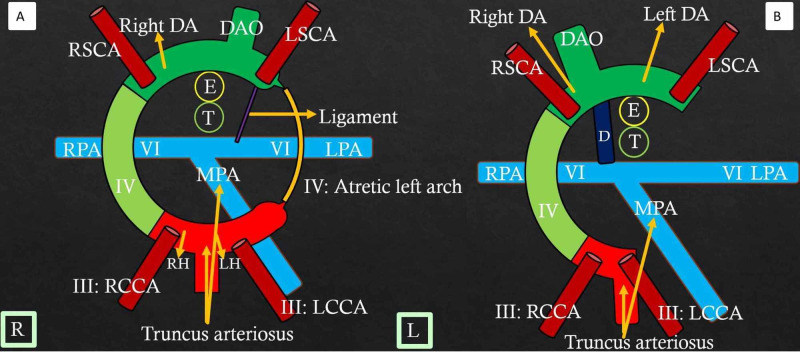
Double arch with atresia between the LCCA and LSCA - mimics right arch with the aberrant left subclavian artery Color diagram (A) shows the atretic cord (yellow) between the left common carotid and left subclavian artery that completes the vascular ring on the left side. This presentation resembles the right aortic arch with the aberrant left subclavian artery (B) where there is persistence of the right fourth arch (light green), right dorsal aorta, and dissolution of the left fourth arch. The left dorsal aorta persists behind the esophagus and gives rise to the left subclavian artery with evidence of the right ductus and right-sided descending aorta. III, IV, and VI refer to the third, fourth, and sixth branchial arches. RSCA: Right subclavian artery; LSCA: Left subclavian artery; MPA: Main pulmonary artery; RPA: Right pulmonary artery; LPA: Left pulmonary artery; RCCA: Right common carotid artery; LCCA: Left common carotid artery; Right DA: Right dorsal aorta; Left DA: Left dorsal aorta; DAO: Unpaired fused dorsal aorta; D: Ductus; T: Trachea; E: Esophagus. R and L in square boxes mention right and left orientation

Here, the atretic DAA anatomy (Figure 7A) resembles the right aortic arch with an aberrant left subclavian artery (Figure 7B) (Video 1) [3-4].

DAA With Atresia Between the Ductus and Left Subclavian Artery

**Figure 8:**
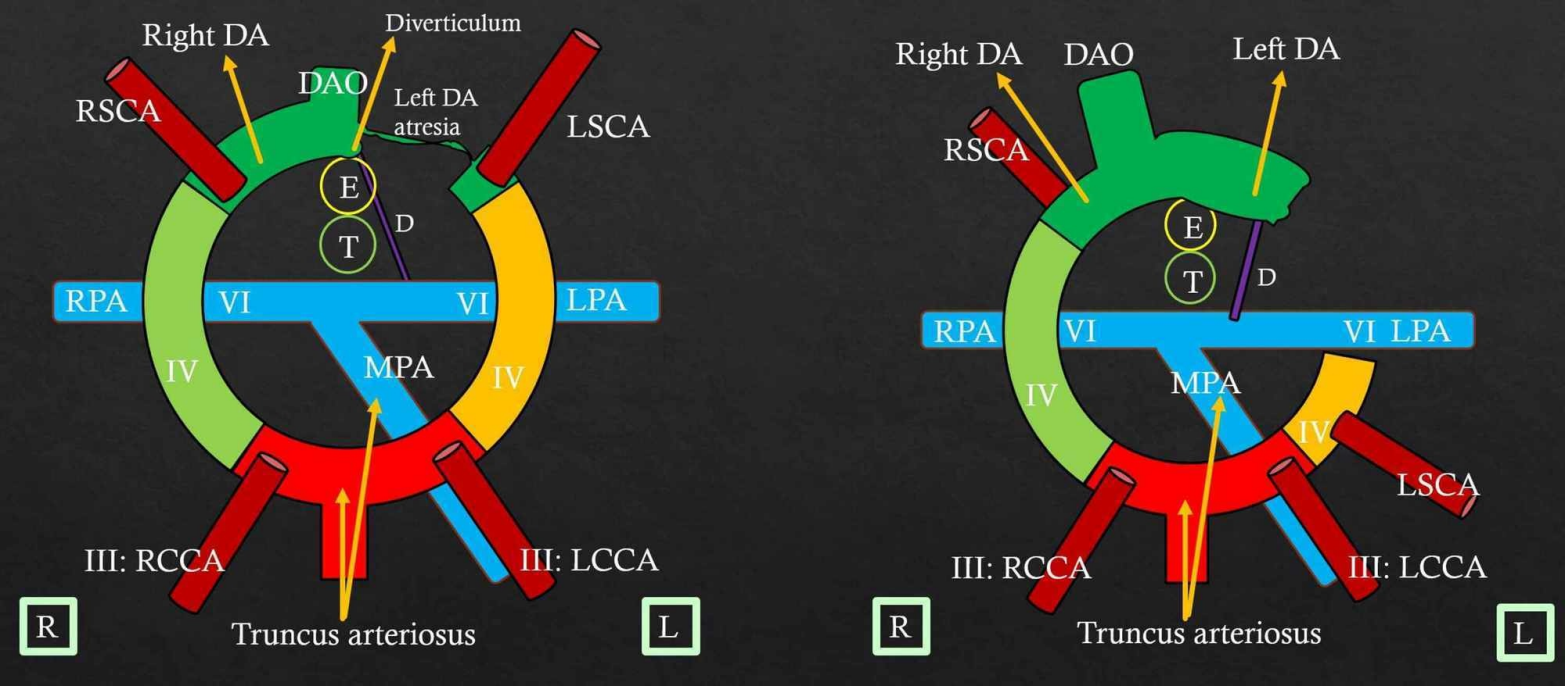
Double arch with atresia between left ductus and left subclavian artery - mimics right aortic arch with Type 2 mirror image branching Color diagram (A) shows atresia in the left dorsal aorta segment between the left ligamentum arteriosum and left subclavian arteries. The vascular ring is completed by the atretic left arch and left ligamentum arteriosum. Here, the ductus joins the descending aortic diverticulum. This appearance of an atretic double arch resembles the right aortic arch with Type 2 mirror image branching (B) in which there is dissolution of the left fourth arch. However, there is persistence of the left dorsal aorta that runs a retroesophageal course and gives rise to the diverticulum that joins the left ductus completing the vascular ring on the left side. RH: Right horn of aortic sac; LH: Left horn of aortic sac; RSCA: Right subclavian artery; LSCA: Left subclavian artery; MPA: Main pulmonary artery; RPA: Right pulmonary artery; LPA: Left pulmonary artery; RCCA: Right common carotid artery; LCCA: Left common carotid artery; Right DA: Right dorsal aorta; Left DA: Left dorsal aorta; DAO: Unpaired fused dorsal aorta; Ligament: Ligamentum arteriosum; D: Ductus; T: Trachea; E: Esophagus. R and L in square boxes mention right and left orientation

In such patients, atresia involves the left dorsal aorta segment between the left ductus and left subclavian arteries (Figure 8A). This appearance resembles an extremely rare aortic arch anomaly viz. the right aortic arch with Type 2 mirror image branching (Figure 8B). Differentiation between the right aortic arch with Type 2 mirror image branching and DAA with atresia distal to the left common carotid artery is less relevant clinically, as both results in the formation of the complete vascular ring [3].

Management

DAA is managed by the surgical division of the non-dominant arch in patients with respiratory or esophageal symptoms [20]. 3D images are helpful to accurately characterize the site of arch dominance, as thoracotomy is performed on the side of the non-dominant arch. It is also critical during surgery to actively look for the ductus/ligamentum arteriosum and resect it, as the patient may remain symptomatic if only the arch is divided and the ductus or ligamentum arteriosum is left intact.

## Conclusions

An atretic double aortic arch is an uncommon cause of a complete vascular ring. An echocardiogram is frequently limited in assessing the atretic double aortic arch and its branches due to the suboptimal window and dependence on patient cooperation. Non-gated CTA with three-dimensional visualization is an excellent technique for the visualization of atretic DAA and differentiates it from its close mimics. A symmetric four-vessel sign at the thoracic inlet, posterior course of a patent segment of the atretic left arch, and presence of a diverticulum at the descending aorta are the diagnostic clues that differentiate atretic DAA from the right aortic arch with mirror image branching or the right arch with an aberrant left subclavian artery.
